# Effects of maturation on myotonometric parameters and their predictors of athletic performance in elite youth soccer players

**DOI:** 10.1038/s41598-024-63224-3

**Published:** 2024-05-29

**Authors:** Alberto García-Santamaría, Cristian Abelairas-Gómez, Samuel Carrera, Alexis Padrón-Cabo, Ezequiel Rey

**Affiliations:** 1 Faculty of Education and Sport Sciences, University of Vigo, Pontevedra, Spain; 2 Faculty of Education Sciences, Universidade de Santiago de Compostela, Santiago de Compostela, Spain

**Keywords:** Physiology, Sensors and probes, Musculoskeletal system, Muscle, Skeletal muscle

## Abstract

The aim of the present study was to investigate the variations in individual muscle stiffness across different maturation stages (i.e., peak height velocity [PHV]) in elite youth soccer players and to explore the associations between lower limb muscle stiffness and performance in sprinting (10, 20, and 40 m sprint), maneuverability (9–3-6–3-9 m sprint test), and jumping (countermovement jump [CMJ]). A total of 131 elite youth soccer players aged 12–18 years, volunteered to participate in the study and were divided into pre-PHV (n = 21), mid-PHV (n = 33), and post-PHV (n = 80). Muscle stiffness of the rectus femoris (RF) and biceps femoris (BF) muscles was assessed using a MyotonPRO. Results showed that players in the pre-PHV stage had lower stiffness in the BF and RF muscles compared to mid-PHV (*p* < 0.001; effect size [ES] = moderate to large) and post-PHV players (*p* < 0.001; ES = moderate to large). It was also observed that the mid-PHV group had lower stiffness levels in their RF muscle compared to the post-PHV group (*p* < 0.001; ES = small). Significant correlations were found between BF and RF stiffness and sprint (*p* < 0.001) and maneuverability (*p* < 0.001) performance. RF stiffness showed a significant positive correlation with CMJ (*p* < 0.05), suggesting that greater lower body stiffness is beneficial for athletic performance in youth soccer players. The findings highlighting the importance of considering training methods that increase muscular stiffness, particularly in relation to the RF muscle, to optimize athletic performance.

## Introduction

Talent identification among soccer players from a young age and their subsequent development process into elite athletes has become pivotal strategies for achieving sporting success^1^. This selection process is inherently intricate and contingent upon the interplay of physical, technical-tactical, and psychological factors^2^. Specifically, within contemporary soccer, neuromuscular capabilities assume a significant role, driven by the increasing frequency of high-intensity actions observed in professional matches^3^. In this context, recent findings by Deprez et al.^4^ have highlighted that athletes demonstrating pronounced explosiveness were predisposed to securing professional contracts and, subsequently, increased playing time upon attaining professional status. Consequently, it becomes essential to delineate players’ neuromuscular profiles to enhance the precision of talent identification in soccer.

Within this framework, prior research has revealed that using muscular stiffness measurements for evaluating neuromuscular status could potentially offer a more discerning metric for identifying an athlete's status and assessing their performance^5^. The contractile characteristics of muscles appear to be contingent upon the process of pubertal maturation. The human organism undergoes substantial morphological and endocrinological alterations during the transition from the juvenile stage to full-fledged adulthood. The contractile properties of muscles may be subject to modulation through the action and concentration of endogenous hormones, specifically testosterone and estrogen, which exert a pivotal influence on muscular development^6^. Throughout the course of childhood, research has demonstrated an increase in musculoskeletal stiffness from the age of 7 years to adulthood in humans^7^. Nevertheless, ongoing discussions persist regarding whether this increase is predominantly contingent on age or is influenced by other variables, such as age-related enhancements in body mass and force production capabilities^6^. However, despite the potential for enhanced athletic performance and links to injury, assessments of lower-body musculature stiffness are infrequently performed in elite soccer academies, perhaps because of perceived practical constraints and intricacies^8^.

In light of this information, there is a need to gain a deeper understanding of the neuromuscular changes that occur with age and biological maturation within sporting cohorts^9^. Surprisingly, little attention has been given to age’s effects on muscle contractile properties in youth soccer players. To date, only one study has analyzed the variations in stiffness levels at different developmental stages of the maturation process in youth soccer players. Specifically, Padrón-Cabo et al.^10^ examined the impact of maturation on the contractile properties of the rectus femoris (RF) and biceps femoris (BF) muscles in youth soccer players, utilizing tensiomyography (TMG) as the assessment method. The study's findings concluded that the maturation status did not exhibit a statistically significant effect on the mechanical and contractile attributes of these muscular structures.

Recently, the MyotonPRO has been developed as a simple, non-invasive, lower-cost, reliable, and user-friendly tool, making it accessible to individuals with less technical expertise^11^. It is an effective method for assessing muscle mechanical properties when compared to other methods, such as TMG^12,13^. MyotonPRO measurements have shown superior relative and absolute reliabilities and high sensitivity to changes compared to TMG^14–16^.

Consequently, the primary aim of the current cross-sectional study was to examine potential variations in individual muscular stiffness across different developmental stages of maturation in soccer players aged 12 to 18 years. The secondary aim was to explore the associations between lower limb muscular stiffness and performance in sprinting, maneuverability, and jumping.

## Methods

### Design

This cross-sectional comparative study was conducted with the aim of investigating differences in the contractile properties of lower limb muscles concerning both the age and maturation status of youth elite soccer players. Additionally, the study sought to explore the relationships between these contractile properties and physical performance. Based on soccer talent identification previous evidence (1), physical performance assessments included tests of linear speed (10 m, 20 m, and 40 m sprints), maneuverability (9-3-6-3-9 m sprint test), and vertical jump height using the countermovement jump (CMJ) test. This study was carried out during the second phase of the in-season period, from February to March. For each age group, all the tests were performed during an experimental session, in the same order (with the myotonometric assessment first, followed by the physical performance tests), using the same testing equipment, measurement protocols, and experienced evaluators. To mitigate potential impacts of fatigue on MyotonPro and physical performance measurements, all participating athletes underwent testing sessions after a recovery interval of no less than 72 h. These testing sessions were consistently conducted in an outdoor artificial pitch turf during the afternoon between 17:00 and 19:00 h and maintained a uniform environmental setting, characterized by temperatures ranging from 19 to 22 °C and relative humidity levels of 52% to 57%.

### Subjects

A total of 131 elite youth soccer players (mean age 15.53 ± 2.17 years; mean height 169.33 ± 13.16 cm; mean weight 59.34 ± 13.68 kg) with a chronological age range spanning from 12 to 18 years volunteered to participate in this study (Table 1). The participants were recruited from the same academy affiliated with a Spanish professional team. These players were drawn from six age categories, encompassing U13, U14, U15, U16, U17, and U18. Players’ characteristics are presented in Table 1. All players actively participated in 90-min training sessions on artificial turf for ten months of the year. These sessions included a comprehensive blend of physical conditioning, technical skill development and tactical drills. All participants possessed between 6 and 11 years of soccer training experience.

**Table 1:** Players characteristics by age group.

Age category	Age (years)	Height (cm)	Body mass (kg)	Age at PHV	Number of sessions per week
U18	17.43 ± 0.37	178.4 ± 6.5	70.3 ± 5.1	14.15 ± 0.42	5
U17	16.23 ± 0.42	177.1 ± 9.8	66.1 ± 4.9	13.76 ± 0.38	4
U16	15.18 ± 0.45	171.6 ± 9.8	58.1 ± 5.9	13.70 ± 0.52	4
U15	14.41 ± 0.33	163.2 ± 9.8	53.6 ± 8.3	13.74 ± 0.49	4
U14	13.39 ± 0.29	156.3 ± 10.9	43.8 ± 7.9	14.15 ± 0.64	4
U13	12.35 ± 0.41	149.2 ± 8.8	38.4 ± 7.4	13.53 ± 0.38	4

U18 = under 18; U17 = under 17; U16 = under 16; U15 = under 15; U14 = under 14; U13 = under 13; U12 = under 12; PHV = peak height velocity.

The inclusion criteria stipulated that the youth players should be free from any injuries in the previous 6 months and that they played in outfield positions, excluding goalkeepers from subsequent analysis. An a priori power analysis was performed using G*Power software (version 3.1.9.2, Universität Kiel, Düsseldorf, Germany)^17^. For power calculations (1-β), the effect size was set at 0.5, assuming a type I error of 0.05 and a type II error of 0.20 (equating to 80% statistical power) for one-way analysis of variance (ANOVA). This analysis indicated that a minimum of 14 players per group would be sufficient to achieve the desired statistical power. Participants were categorized into three maturity groups according to the maturity offset: pre-PHV (< − 1.0 year), mid-PHV (− 0.99 to 0.99 years), and post-PHV (> 1 year) using the alternative model for boys by Moore et al.^18^ (Table 2). Each player's participation in the study was contingent upon the informed consent form being signed by a parent or legal guardian. The informed consent form, institutionally approved, outlined the benefits and potential risks associated with the research, and this information was provided to the participants' legal representatives prior to obtaining their consent. The research protocol received approval from the Local Ethics Committee (University of Vigo; Reference 19-0320) and was conducted in accordance with the Code of Ethics of the World Medical Association, as defined in the Declaration of Helsinki.

**Table 2:** Characteristics of participants (mean ± SD) according to the maturational groups.

	Pre-PHV (n = 21)	Mid-PHV (n = 33)	Post-PHV (n = 80)
Age (years)	12.2 ± 0.4	13.7 ± 0.6	16.6 ± 1.1
Heigth (cm)	147.8 ± 7.9	161.3 ± 8.6	176.6 ± 6.1
Body mass (kg)	37.5 ± 6.0	50.2 ± 8.7	67.0 ± 6.8
BMI	17.0 ± 1.6	19.1 ± 2.0	21.4 ± 1.6
APHV	14.0 ± 1.6	13.7 ± 0.5	14.0 ± 0.5
Maturity offset	− 1.8 ± 1.4	0.1 ± 0.5	2.6 ± 0.7

APHV: age at peak height velocity; BMI: body mass index; PHV: peak heigh velocity.

### MyotonPro measurement protocol

The evaluation focused on the RF and BF muscles due to their crucial roles in high-intensity running and technical actions in soccer^19^. Both RF and BF were assessed by a MyotonPRO digital palpation device (Myoton AS, Tallinn, Estonia). To assess the RF muscle, players assumed a supine posture with their hips in a neutral position and their knees fully extended. The test landmark was positioned at a point two-thirds of the distance between the anterior superior iliac spine and the superior pole of the patella^20^. To evaluate the BF muscle, participants were placed in a prone position with their hands resting flat on both sides of their bodies, and their feet naturally hanging off the edge of the bed. The test landmark was located at a point 50% of the distance from the isocenter to the lateral epicondyle of the femur^21^.

The assessment procedure involved applying a 3-mm diameter probe's tip perpendicularly to the skin surface above the muscle. A brief (15 ms), low-force (0.4 N) mechanical impulse was applied, resulting in a consistent compression of 0.18 N on the subcutaneous superficial tissues. This compression was then transmitted to the underlying muscle, and the subsequent damped oscillation of the muscle was recorded using an accelerometer. Various parameters were measured, including oscillation frequency (Hz), logarithmic decrement (arbitrary units), dynamic stiffness (N/m), mechanical stress relaxation time (ms), and creep (Deborah number). Frequency characterizes the intrinsic tension of the muscle in its resting state without voluntary contraction, providing insights into muscle tone. Stiffness reflects the muscle's resistance to contraction or external forces that deform its initial shape. Logarithmic decrement gauges muscle elasticity concerning its ability to recover its initial shape following contraction or the removal of external forces. Relaxation time denotes the time required for the muscle to restore its shape after deformation caused by voluntary contraction or the removal of external forces. Creep signifies the gradual elongation of the muscle over time when subjected to constant tensile stress^22^. The mean value of five consecutive measurements at each anatomical site was used for analysis^23^. If the coefficient of variation exceeded 3%, the measurement was repeated.

#### m, 20 m, and 40 m sprint test

Sprint time was assessed using a dual infrared reflex photoelectric cell system (Witty, Microgate, Bolzano, Italy). The photoelectric cells were securely mounted on tripods at a height of 0.9 m and positioned in pairs with a separation of 1 m between them. Prior to the start of each sprint, all participants initiated from a stationary position, ensuring their leading foot was situated 0.5 m away from the initial timing gate. They were explicitly instructed to exert maximal effort during each sprint trial. The testing protocol allowed for 3 attempts, with a 2-min inter-trial recovery period. The superior time recorded from the two trials was selected for further analysis. The reliability of the test–retest trials was evaluated using the intraclass correlation coefficients [ICC_(3,1)_], yielding values of 0.92 (95% CI 0.90–0.94), 0.97 (95% CI 0.96–0.98), and 0.98 (95% CI 0.97–0.98) for the 10 m, 20 m, and 40 m sprints, respectively.

### 9-3-6-3-9 m sprint with backward and forward running (SBF)

The SBF was employed to assess maneuverability skills^24^. The evaluation of SBF performance was executed utilizing a photoelectric cell system (Witty, Microgate, Bolzano, Italy). In accordance with the established protocol proposed by Sporis et al.^25^, participants were initiated upon receiving a signal and were required to traverse a series of designated lines over a specified distance. The trajectory began at starting line A and entailed a 9-m sprint to line B, which was demarcated by white lines measuring 3 m in length and 5 cm in width. Upon reaching line B and making contact with it using one foot, participants transitioned from forward running to backward running. Subsequently, a 3-m sprint to line C ensued, prompting a shift from backward running to forward running. After completing a 6-m stretch, participants encountered another transition point (line D) necessitating a 3-m backward run (line E). The sequence culminated in a final transition, followed by a 9-m forward sprint to the finish line (line F). The assessment procedure enabled each participant to undertake three trials, with a 2-min inter-trial recovery period interposed between attempts. The ICC_(3,1)_ for test–retest trials was 0.98 (95% CI 0.96–0.99).

### Vertical jump performance

The countermovement jump (CMJ) was conducted using a force plate (Ergo Jump Bosco System; Globus, Treviso, Italy) following the procedures outlined by Bosco et al.^26^. Each player was allowed 3 trials, with a 1-min rest interval between each attempt. The best trial was selected for subsequent analysis. The countermovement jump commenced from a squat starting position, with the knees flexed at 90 degrees, and the hands placed on the hips. From this initial posture, the soccer players were instructed to flex their knees to a self-selected angle and execute a maximal vertical thrust. Throughout the jump, participants maintained their hands on their hips to eliminate the influence of arm movement. Participants were also directed to maintain a vertical body position during the jump, avoiding any undue lateral or frontal movements, and land with fully extended knees. Any jump that deviated from the prescribed instructions was repeated. The ICC_(3,1)_ for test–retest trials was 0.97 (95% CI 0.95–0.98).

### Statistical analysis

Means and standard deviations for all dependent variables of interest were used as measures of centrality and the spread of data. A one-way analysis of variance (ANOVA) with Bonferroni post hoc contrasts was used to detect differences in the variables of interest between the three maturity groups (Pre-PHV vs. Mid-PHV vs. Post-PHV). Cohen’s *d* was computed to compare effect sizes (ES). ES were classified as trivial (*d* < 0.2), small (0.2 ≤ *d* < 0.5), moderate (0.5 ≤ *d* < 0.8), and large (≥ 0.8). Pearson correlation coefficients were used to determine the strength of relationships between sprint, maneuverability, CMJ, and muscular stiffness, with the strength of relationships classified as follows: < 0.10 (trivial), 0.10 to 0.29 (small), 0.30 to 0.49 (moderate), 0.50 to 0.69 (large), 0.70 to 0.89 (very large), and > 0.90 (nearly perfect)^27^. All statistical analyses were conducted in SPSS Statistics version 25 for Mac (SPSS Inc., Chicago, IL, USA), with statistical significance set at an alpha level of *p* < 0.05.

## Results

### Differences between maturational groups

Table 3 illustrates the comparisons between maturational groups concerning myotonometry mechanical properties, vertical jump, linear sprint, and maneuverability. The one-way ANOVA analysis revealed significant differences in all tests, except for BF decrement (*p* = 0.375). Specifically, significant differences were observed in the frequency (ES = moderate to large), relaxation (ES = moderate to large), creep (ES = moderate to large), and stiffness (ES = moderate to large) of both BF and RF between the Pre-PHV and Mid-PHV groups, as well as between the Pre-PHV and Post-PHV groups. Additionally, a significant increase in RF stiffness was noted from the Mid-PHV to the Post-PHV group (ES = small). Furthermore, there were significant differences in CMJ (ES = large), the 10-m sprint (ES = large), 20-m sprint (ES = large), 40-m sprint (ES = large), and the 9–3-6–3-9 test (ES = large) between the Pre-PHV and Mid-PHV groups, as well as between the Pre-PHV and Post-PHV groups. Additionally, the 10-m sprint (ES = large), 20-m sprint (ES = large), 40-m sprint (ES = large), and the 9–3-6–3-9 test (ES = large) showed significant differences between the Mid-PHV and Post-PHV groups.

**Table 3:** Differences between maturational groups on myotonometry mechanical properties, vertical jump, linear sprint, and agility.

	Maturational groups			One-way ANOVA		Effect size (95% CI)		
	Pre-PHV (*n* = 21)	Mid-PHV (*n* = 33)	Post-PHV (*n* = 80)	F-value	*p*-value	Pre-PHV vs. Mid-PHV	Mid-PHV vs. Post-PHV	Pre-PHV vs. Post-PHV
BF frequency (Hz)	13.40 ± 1.66*	14.55 ± 1.20	14.65 ± 1.57	5.836	0.004	0.82 (0.25; 1.39)	0.06 (− 0.33; 0.47)	0.78 (0.29; 1.27)
BF decrement (au)	1.25 ± 0.25	1.32 ± 0.17	1.30 ± 0.15	0.989	0.375	0.34 (− 0.20; 0.89)	0.12 (− 0.27; 0.53)	0.28 (− 0.19; 0.76)
BF relaxation (ms)	20.53 ± 2.86*	18.25 ± 1.88	17.99 ± 2.28	10.341	< 0.001	0.98 (0.41; 1.56)	0.11 (− 0.28; 0.52)	1.05 (0.55; 1.55)
BF stiffness (N⋅m^-1^)	228.6 ± 54.3*	275.7 ± 36.4	284.4 ± 46.2	12.520	< 0.001	1.06 (0.48; 1.64)	0.27 (0.12; 0.67)	1.15 (0.65; 1.66)
RF frequency (Hz)	12.76 ± 0.61*	13.50 ± 1.13	13.95 ± 1.46	7.439	0.001	0.76 (0.20; 1.33)	0.32 (− 0.08; 0.73)	0.89 (0.39; 1.38)
RF decrement (au)	1.17 ± 0.18**	1.29 ± 0.19	1.36 ± 0.25	6.153	0.003	0.64 (0.08; 1.20)	0.29 (− 0.10; 0.70)	0.79 (0.30; 1.29)
RF relaxation (ms)	20.57 ± 1.36*	19.46 ± 1.98	18.81 ± 1.95	7.480	0.001	0.62 (0.06; 1.18)	0.33 (− 0.07; 0.73)	0.95 (0.45; 1.45)
RF stiffness (N⋅m^-1^)	224.3 ± 24.2*	246.0 ± 37.4**	262.8 ± 42.6	8.791	< 0.001	0.66 (0.09; 1.22)	0.45 (0.04; 0.85)	0.96 (0.47; 1.45)
CMJ (cm)	28.8 ± 5.2*	39.7 ± 7.0	41.0 ± 6.4	30.027	< 0.001	1.71 (1.07; 2.34)	0.19 (0.21; 0.60)	1.97 (1.42; 2.52)
10 m sprint (s)	2.25 ± 0.18*	2.00 ± 0.14**	1.89 ± 0.13	52.093	< 0.001	1.59 (0.97; 2.22)	0.82 (0.40; 1.24)	2.54 (1.94; 3.13)
20 m sprint (s)	3.86 ± 0.23*	3.39 ± 0.18**	3.00 ± 0.19	168.583	< 0.001	2.34 (1.63; 3.04)	2.08 (1.59; 2.57)	4.32 (3.56; 5.09)
40 m sprint (s)	7.02 ± 0.49*	6.11 ± 0.38**	5.43 ± 0.31	170.515	< 0.001	2.13 (1.45; 2.81)	2.05 (1.56; 2.53)	4.49 (4.71; 5.27)
9–3-6–3-9 (s)	8.88 ± 0.56*	8.14 ± 0.39**	7.59 ± 0.49	62.055	< 0.001	1.59 (0.97; 2.22)	1.18 (0.75; 1.62)	2.55 (1.95; 3.15)

BF: biceps femoris muscle; RF: rectus femoris muscle; CMJ: countermovement jump; au: arbitrary units; dn: Deborah number; PHV: peak height velocity.

*Different from the mid-PHV and post-PHV groups (p < 0.001).

**Different from the post-PHV group (p < 0.005).

### Relationships between physical test performance and muscle stiffness

Selected bivariate correlations between muscle stiffness and physical performance are presented in Table 4. BF stiffness exhibited significant negative correlations with the 10-m sprint (small), 20-m sprint (moderate), 40-m sprint (moderate), and the 9–3-6–3-9 test (moderate). Similarly, RF stiffness demonstrated significant negative correlations with the 10-m sprint (moderate), 20-m sprint (small), 40-m sprint (moderate), and the 9–3-6–3-9 test (moderate). Furthermore, RF stiffness displayed a significant positive correlation with CMJ (small).

**Table 4:** Correlation results between rectus and biceps femoris and physical performance assessments.

Variable	BF stiffness	RF stiffness
10 m sprint	− 0.262*	− 0.374*
20 m sprint	− 0.374*	− 0.296*
40 m sprint	− 0.368*	− 0.324*
CMJ	0.146	0.224**
9–3-6–3-9	− 0.356*	− 0.329*

CMJ: countermovement jump; BF: biceps femoris; RF: rectus femoris.

*Significant correlation (*p* < 0.001).

**Significant correlation (*p* < 0.05).

## Discussion

The study aimed to investigate variations in individual muscular stiffness across different developmental stages in soccer players aged 12 to 18 years. It also aimed to explore the associations between lower limb muscular stiffness and sprinting, maneuverability, and jumping performance. The key findings indicated that players in the pre-PHV stage had lower stiffness in the BF and RF muscles compared to mid-PHV and post-PHV players. Additionally, it was observed that the mid-PHV group exhibited lower stiffness levels in their RF muscle compared to the post-PHV group. Relationships between the stiffness of BF and RF and sprint and maneuverability performance were identified. Finally, RF stiffness displayed a significant positive correlation with CMJ. The implications of these findings have a bearing on the formulation of training recommendations for young footballers. The results indicate that practitioners, such as trainers and coaches, should examine techniques designed to enhance muscular stiffness, particularly in relation to the RF muscle.

As expected, the findings of the present study showed that maturation results in significant improvements in sprint, maneuverability, and vertical jump performance. In particular, sprint and maneuverability performance were better in post-PHV compared to both pre-PHV and mid-PHV, as well as in mid-PHV compared to pre-PHV. In addition, CMJ performance was also superior in mid-PHV and post-PHV compared to pre-PHV, with no differences between these last two groups. These findings contribute significantly to the existing body of knowledge and are consistent with prior research regarding the influence of maturation on physical performance among young soccer players, underscoring the positive impact of maturation on their physical capacities^28,29^. Contrary to certain previous evidence suggesting a progressive decline in change of direction ability in soccer, our results challenge this notion, highlighting the potential for continued enhancement in this aspect with maturation^30^.

This study represents the first attempt to investigate the mechanical properties of youth soccer players as a function of their maturity status using myotonometric measurements. The results showed that the maturation of soccer players significantly affects muscle tone, stiffness, elasticity and relaxation, but with slight differences between RF and BF. Passive tone (frequency) and stiffness of the BF muscle increased as the players matured (from pre-PHV to mid- and post-PHV), while relaxation decreased. On the other hand, pre-PHV players had significantly lower levels of muscle tone, elasticity, and stiffness in the RF compared to their mid-PHV and post-PHV counterparts. In addition, mid-PHV individuals had lower RF stiffness than post-PHV individuals. Based on the results of the present study, the application of myotonometry in youth soccer players requires consideration of the maturational status of the individual player. Additionally, these results provide valuable myotonometry reference information for elite male youth soccer players, which may be critical in monitoring player development and talent identification.

The observed increases in both the active and passive stiffness components of RF and BF can be attributed to various underlying mechanisms such as increases in muscle length, cross-sectional area, or intramuscular filaments in response to the maturation process^31^. The lack of myotonometric values for the present cohort of youth soccer players precludes direct comparisons with previous studies. Nevertheless, our findings are consistent with those of Meyers et al.^32^, who reported a significant age-related increase in absolute vertical stiffness in youth soccer players aged U12 to U16. Furthermore, Rumpf et al.^33^ demonstrated an increase in leg stiffness from pre- to post-PHV in a cohort of seventy-four physically active males aged 8 to 16 years. However, it is important to note that both of the above studies used segmental stiffness assessment rather than individual muscle stiffness assessment. To date, only one recent study by Padrón-Cabo et al.^10^ has investigated the effect of maturation on individual muscle stiffness using TMG. The study conducted on 121 elite youth soccer players revealed non-significant effects of maturation status on RF and BF stiffness, contraction time, and contraction velocity. Equally, Sanchez-Sanchez et al.^34^ also reported that RF and BF stiffness and contraction time are not age-specific in youth soccer players from U-14 to U-18 age categories. The discrepancies observed between the present results and Padrón-Cabo et al.^10^ and Sanchez-Sanchez et al.^34^ may be partially attributed to the superior sensitivity and reliability of myotonometry in detecting contractile and mechanical changes compared with TMG^15^. Therefore, it is recommended to consider using myotonometry to assess muscle properties in youth soccer players while taking into account the maturation status of individual players.

The relationship between stiffness and physical performance is complex and multifaceted, influenced by various factors, including age, sex, muscle-specific characteristics, and activity levels. The positive association between increased musculoskeletal stiffness and elite athletic performance during fast stretch-shorten cycle activities has been extensively documented in prior research^35^. In congruence, the current findings revealed that stiffness measurements collected by a myometer under passive conditions appear to be related to dynamic muscle activities in youth soccer players. Concretely, significant positive correlations were determined between myometer stiffness of RF and BF and 10-m sprint, 20-m sprint, 40 m-sprint, and SBF maneuverability test. These results are in keeping with those of Kalkhoven and Watsford^36^ who found that individual muscle stiffness of RF, assessed with Myoton-Pro, was positively correlated with 10-m, 20-m, 30-m, 40-m, 50-m, and 60-m sprint and 5–0-5 agility test in sub-elite male soccer players. Similarly, Pruyn et al.^37^ reported a significant positive correlation between medial gastrocnemius and soleus stiffness, assessed with myometer, and 10-m sprint and 5–0-5 agility test in netball female athletes. One potential mechanism for explaining the role of RF and BF in enhancing sprint and maneuverability performance may be associated with an increased rate of force development, which is usually linked to higher stiffness values^36,38^.

Several studies have explored the correlation between stiffness and jumping ability, yet this relationship remains largely uncertain. The current study found a significant positive correlation between RF stiffness and CMJ performance, but no relationship between BF stiffness and jump ability. These findings could be attributed to the significant contribution of the RF in generating force during CMJ, as opposed to the BF (with a ratio of 35% MVIC to 15% MVIC, respectively)^39^. Contrasting with these results, Kalkhoven and Watsford^35^ failed to report significant correlations between RF stiffness and CMJ performance among professional male soccer players. These conflicting findings highlight the complexity of the relationship between muscle stiffness and jumping ability and suggest that additional factors may influence this association.

Understanding the relationship between the performance of dynamic skills and a modifiable mechanical muscle property such as stiffness is critical as it may impact future conditioning regimens for team sport athletes. Given that stiffness is a modifiable mechanical factor, the results of this investigation provide pertinent insights for players, coaches, and conditioning staff regarding the improvement of lower body stiffness to optimize athletic performance. Additionally, consistent with the results of the present study and supporting literature, it appears that greater lower body stiffness is advantageous for both males and females when SSG movements, which is particularly relevant in soccer where athletes frequently perform maximal and repetitive jumping actions. Consequently, strategies such as plyometric training or strength training could potentially improve lower body stiffness and should be considered for implementation whenever feasible^8^.

This study design provides valuable insights into variations in muscle stiffness and its associations with performance in youth soccer players, including the innovative approach of investigating the contractile properties of lower limb muscles in youth soccer players at different stages of maturation using MyotonPRO, a reliable and user-friendly tool for assessing muscle mechanical properties. However, it is important to acknowledge and address the limitations of the present study to ensure the robustness and applicability of the findings. First, the study focused on the contractile properties of only two specific muscles, RF and BF, which may not fully capture the overall neuromuscular profiles of the players. In addition, the study did not account for other potential factors that may influence neuromuscular development, such as diet, previous injuries, or specific training regimens, which may limit a comprehensive understanding of players' neuromuscular status. Furthermore, the manuscript lacks a thorough discussion of potential confounding variables that could affect the results, such as the players' level of physical activity outside of soccer training or their psychological factors. Finally, the study did not examine the long-term implications of the observed differences in muscle stiffness across age groups and developmental stages, which may limit the practical application of the findings for talent identification and player development programs. Future studies could address these limitations by using longitudinal designs, including control groups, controlling for potential confounders more comprehensively, including female players, and assessing a broader range of lower limb muscles.

## Conclusions

The study on variations in individual muscular stiffness across different developmental stages in soccer players aged 12 to 18 years revealed significant differences in muscle stiffness levels between pre-PHV, mid-PHV, and post-PHV players. The study also identified significant positive correlations between RF stiffness and CMJ performance, as well as between BF and RF stiffness and sprinting and maneuverability performance. This suggests that practitioners, including trainers and coaches, should prioritize techniques to improve muscle stiffness, particularly in relation to the RF muscle.

## Data Availability

The datasets used and analysed during the current study are available from the corresponding author (zequirey@uvigo.es) on reasonable request.
